# Postgraduate Education in Disaster Health and Medicine

**DOI:** 10.3389/fpubh.2015.00185

**Published:** 2015-08-10

**Authors:** Khalid Yousif Ahmed Algaali, Ahmadreza Djalali, Francesco Della Corte, Mohamed Ahmed Ismail, Pier Lugi Ingrassia

**Affiliations:** ^1^CRIMEDIM – Research Centre in Emergency and Disaster Medicine and Computer Science Applied to Medical Practice, Università Degli Studi del Piemonte Orientale, Novara, Italy; ^2^Department of Clinical Science and Education, Karolinska Institutet, Stockholm, Sweden; ^3^Sudanese Resuscitation Council, Khartoum, Sudan

**Keywords:** education, disaster, health, medicine, postgraduate

## Abstract

**Introduction:**

Education is key to effective disaster management. This study reviews several postgraduate educational programs in disaster medicine.

**Methods:**

This cross-sectional study was conducted in two stages between October 2011 and February 2012. An online search was completed, followed by a web-based survey to collect information on key aspects of the identified programs.

**Results:**

Thirty-four programs were identified worldwide. Public health was the main focus in 84% of these. E-learning was the preferred mode of instruction in 25% of cases. Most programs were accredited either nationally or internationally. Tuitions fees were the main source of funding.

**Conclusion:**

There is a dearth of postgraduate training programs in disaster health and medicine. This applies especially to Asia, which is also the most vulnerable area. Educational provision must be strengthened in Asia and in low- and middle-income countries to enhance capacity building in the health management of disasters.

## Introduction

Disasters adversely affect communities in terms of health, infrastructures, and environment, with consequences ranging from death, physical injuries, and disability to psychosocial stress. The impact of disasters can be avoided or reduced by adopting risk management measures. These should be implemented by building on local capacities and taking appropriate actions with regards to planning, education, and training (1).

Education and training are necessary for health professionals to gain knowledge and develop those skills that make effective disaster management possible (2, 3). Toward this, several programs have been implemented, and the World Association for Disaster and Emergency Medicine (WADEM) has formulated a standardized international perspective for education and training in disaster medicine and health. These facts notwithstanding, there continues to be a reported gap in competency-based training in disaster medicine (2–6).

A standardized training program should be multidisciplinary and offer both core and elective subjects. Ideally, it should also have a modular approach, include supervised practical training, and generally be geared toward competency-building. It could be implemented as a short-term course or as an academic educational program leading to a master’s or Ph.D. degree (4, 7–9). Long-term training programs have a comprehensive curriculum, certified course content, set educational goals, and might thus be considered more standardized than short courses.

This study focuses on postgraduate disaster medicine education programs at master’s level or equivalent, with the aim to describe and review current provision.

## Methods

This cross-sectional study was conducted in two stages between October 2011 and February 2012. First, we completed an online search to identify postgraduate training programs in disaster health. After this, we prepared a survey to collect information on key aspects of those programs. We sent a web-based survey request followed by a reminder email, with 30% expected retrieval rate (10).

Inclusion criteria were: (1) academic disaster or emergency management programs with a focus on medicine or health; (2) master’s degree level or equivalent; and (3) English-medium instruction.

Exclusion criteria were: (1) Ph.D. programs and (2) non-degree programs (e.g., training courses and workshops).

### Data collection procedures

We did a Google, Bing and Yahoo search for “postgraduate,” “program,” “master,” “degree,” “academic,” “emergency,” “disaster,” “crisis,” “humanitarian,” “medicine,” “health,” and “diploma,” looking for subject-related courses. The search was conducted and checked by two independent researchers. A structured email questionnaire was sent to program directors to collect relevant information, including: type of qualification, curriculum scope and content focus, institutional affiliation, mode of delivery, duration, whether the program is awarded by thesis, academic credit system, program accreditation, number of participants, tuition fees, funding bodies (if any), availability of financial aid to students.

## Results

**Figure 1:**
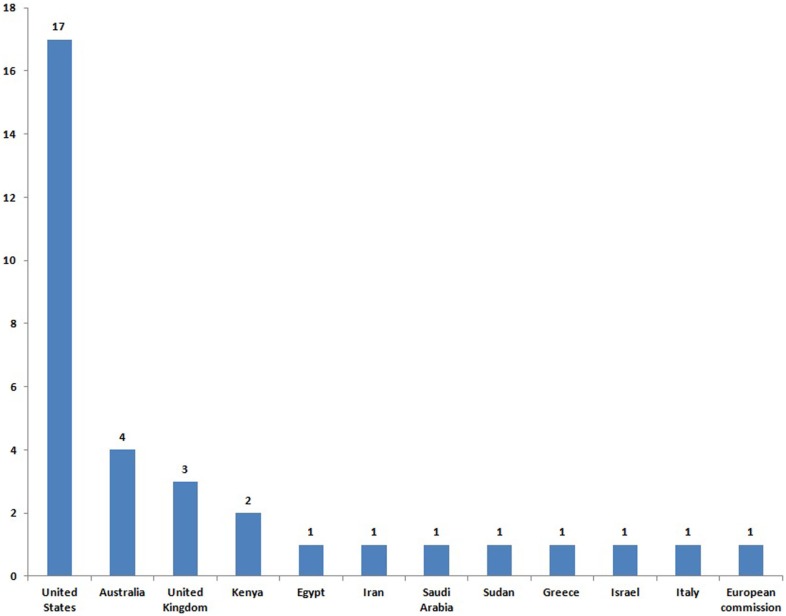
**Distribution of 34 identified postgraduate programs in emergency and disaster health and medicine in 11 countries and European commission**.

A total of 34 postgraduate programs in disaster medicine were found (Table 1). All but one are delivered in 11 countries and the one that is not is run by the European Commission (Figure 1). If we consider WHO regional offices, 50% (=17) of programs were based in the United States and South America, and none was offered in south-east Asia. Of the 34 program directors, 26 agreed to participate in the survey (response rate = 76%). Nineteen programs met the inclusion criteria; the remaining seven did not and were excluded.

**Table 1:** **Identified postgraduate programs in emergency and disaster health and medicine**.

Country	Program title	Institution and URL
Kenya	MPH – Disaster Management	Kenyatta University (http://www.ku.ac.ke/schools/health/images/stories/docs/masters_publichealth.pdf)

Kenya	Master of Public Health – Disaster Management and Preparedness	Moi University (http://www.riskreductionafrica.org/wp-content/uploads/2014/12/Academic-Page-Moi-Univeristy.pdf)

United States	Master of Science in Nursing Administration and Emergency Management	Arkansas Tech University (http://www.atu.edu/academics/catalog-graduate/programs/ms_sci_nur_adm.html)

United States	M.S. in Healthcare Emergency Management	Boston University (http://www.bumc.bu.edu/bmcm/)

United States	Master of Public Health in Forced Migration and Health	Columbia University (http://forcedmigration.columbia.edu/)

United States	MPH Concentration in Health in Crisis and Humanitarian Assistance	Johns Hopkins, Bloomberg School of Public Health (http://www.jhsph.edu/academics/degreeprograms/mph/curriculum/Concentrations/health_in_crisis.html)

United States	Disaster Fellowship	Johns Hopkins University, School of Medicine (http://www.hopkinsmedicine.org/emergencymedicine/fellowship_programs/disaster.html)

United States	International Emergency and Public Health Fellowship	Johns Hopkins University, School of Medicine (http://www.hopkinsmedicine.org/emergencymedicine/fellowship_programs/international_ public_health.html)

United States	Master of Professional Studies in Homeland Security – Public Health Preparedness Option	Pennsylvania State University (http://www.worldcampus.psu.edu/degrees-and-certificates/homeland-security-public-health-preparedness)

United States	M.S. in Disaster Medicine and Management	Philadelphia University (http://www.philau.edu/disastermed/)

United States	Master of Science in Biosecurity and Disaster Preparedness	Saint Louis University (http://www.slu.edu/biosecurity-and-disaster-preparedness-ms)

United States	Master of Public Health in Biosecurity and Disaster Preparedness	Saint Louis University (http://www.slu.edu/college-for-public-health-and-social-justice/academics/public-health-degrees/master-of-public-health/biosecurity-and-disaster-preparedness)

United States	Master of Public Health (MPH) in Disaster Management	Tulane University (http://www.sph.tulane.edu/publichealth/ehs/mph.cfm)

United States	International Disaster Medical Sciences Fellowship	University of California Irvine (http://escholarship.org/uc/item/2006v8f4)

United States	Master of Science in Threat and Response Management	University of Chicago (http://grahamschool.uchicago.edu/mstrm)

United States	M.S. Emergency Health Services	University of Maryland (http://ehs.umbc.edu/graduate)

United States	Disaster Medicine and Emergency Management Fellowship	University of Massachusetts, Medical School (http://www.umassemfellowships.com/disaster.php)

United States	Master of Public Health with a Concentration in Global Disaster Management and Humanitarian Relief	University of South Florida (http://health.usf.edu/publichealth/catalog/2012-2013/programs/03_4_05_mph_gdm.pdf)

United States	Master of Public Health with Emergency Preparedness Concentration	Wright State University (http://www.med.wright.edu/mph)

Egypt	Arab Diploma in Disaster Management for Health Professionals	Arab Institute for Continuing Professional Development (http://www.aicpd.org/cources_details.aspx?id=12)

Iran	MPH with Disaster Concentration	Tehran University of Medical Sciences (http://nihr.tums.ac.ir/disaster/education-2/mph-with-disaster-concentration/)

Saudi Arabia	The Saudi Diploma in Mass Gatherings and Disasters Healthcare	Saudi Commission for Health Specialties (http://english.scfhs.org.sa/en/pages/fellowship_arabia_medicine_crowds.html)

Sudan	Master of Disaster Management and Public Health	Public Health Institute (http://www.phi.edu.sd/master-disastar-managment)

Europe	Erasmus Mundus Master Course in Public Health in Disasters	Universidad de Oviedo International Graduate Centre (http://www.pubhealthdisasters.eu/)

Greece	Master Course in International Medicine-Health Crisis Management	University of Athens (http://crisis.med.uoa.gr/index_en.php)

Israel	International Program in Emergency and Disaster Management	Tel Aviv University (http://www.emergex.tau.ac.il)

Italy	European Master in Disaster Medicine	Università del Piemonte Orientale, Italy and Vrije Universiteit Brussel (http://www.dismedmaster.com/)

United Kingdom	MSc Humanitarian Health Programme Management	Liverpool School of Tropical Medicine (http://www.lstmed.ac.uk/study/courses/humanitarian-health-programme-management)

United Kingdom	MSc International Public Health – Humanitarian Assistance Stream	Liverpool School of Tropical Medicine (http://www.lstmed.ac.uk/study/courses/international-public-health-humanitarian-assistance)

United Kingdom	MSc Disaster Healthcare	University of Glamorgan (http://courses.glam.ac.uk/courses/319-msc-disaster-healthcare)

Australia	Master of Public Health (Biosecurity and Disaster Preparedness)	James Cook University (http://www-public.jcu.edu.au/courses/course_info/index.htm?userText=72504-MPH-BDP)

Australia	Master of Public Health – specialization in Emergency and Disaster Management	Queensland University of Technology (https://www.qut.edu.au/study/courses/graduate-certificate-in-health-science)

Australia	Master of Health Science – Study Areas in Emergency and Disaster Management	Queensland University of Technology (http://www.qut.edu.au/study/courses/master-of-health-science)

Australia	Master of Health Management – Study Area Emergency and Disaster Management	Queensland University of Technology (http://www.qut.edu.au/study/courses/master-of-health-management)

Overall, 74% of programs were master’s degrees, 16% fellowships or board certifications, and 10% postgraduate diplomas. Eighty-four percent of the programs surveyed had a focus on public health and only 16% focused on clinical subjects.

Most courses (75%) were delivered on-site (face-to-face), either full-time (56%) or part-time (19%). The remaining 25% were delivered online. Some programs (21%) in both categories used hybrid formats.

All programs included different types of exercises and drills. These included table-top exercises (63%), functional and full scale exercises (47%), and computer-based exercises (47%). Some programs (37%) also involved deployment to disaster or mass gathering events. Program duration was 12–48 months.

Most programs had a thesis component, either mandatory (42%) or optional (26%); only 32% were not awarded by thesis.

The Australian Qualifications Framework (16%), the European Credit Transfer and Accumulation System (11%), and the US-based credit-hour system (11%) were the most common accreditation schemes. Of the remaining 62%, 25% used other credit systems and 37% had no recognized framework. Sixteen percent of the programs were accredited nationally and 47% internationally; no clarification was given in 37% of cases.

The number of accepted applications varies from 1–2 up to 50 participants per year. Most programs (63%) are delivered by public institutions; the rest were funded by either non-governmental non-for-profit (32%) or private for-profit (5%) bodies. Tuition fees ranged between 1,500 and 59,670 US dollars. For most programs (84%), tuition payments were the main source of funding; in 21% of cases, costs of instruction were covered, fully or partially, by public funding.

Eighty-one percent of programs offered scholarships, 50% offered loans, and 11% offered work-study fellowships as a means of financial support.

## Discussion

This study identified 34 postgraduate programs in disaster medicine. All but one are delivered in 11 countries and the one that is not is run by the European Commission. Since the number of natural and man-made disasters is on the increase (11), the dearth of postgraduate training programs is lamentable worldwide.

Previous studies show different types of deficiencies in disaster-related training (3, 6, 12). The gap is bold in Asia, where most disasters occur and human impact is high (13). Half of the programs are delivered in the United States, which were listed in the top 10 countries for disaster occurrence in 2012 (13). Education is essential to capacity building in the health management of disasters; the need to strengthen provision is therefore real, especially in Asia and in low- and middle-income countries.

This study shows that most programs have a focus on public health-related subjects. This could be because of the range and extent of public health issues during disasters and humanitarian crises. Also, health is a comprehensive word, encompassing a variety of medical subjects, including clinical medicine. A focus on public health is consistent with growing trends in the management of humanitarian crises and epidemics.

Only one-fourth of the evaluated programs are delivered online. E-learning is being implemented more and more frequently in a growing number of subjects. It can help cater for a wide number of trainees in different geographical areas at a relatively low cost. If we add to this that e-learning is also time-flexible and place-flexible, we may conclude that benefits outweigh limitations (3, 14–17). Disaster management is more amenable to e-learning than to traditional training methods, because it helps overcome difficulties with preparing course contents and exercises (14–16).

Blended delivery is an often-suggested alternative. Blended programs incorporate a mix of distance- and traditional, face-to-face training for a richer learning experience. There are several advantages to this approach, including time flexibility, on-going access to course contents, virtual simulations, higher learners’ acceptance and satisfaction, availability of a “diverse array of instructional approaches specifically targeted to the learner audience and the subject matter and skills sets to be delivered” (18, 19).

The programs identified in this study include exercises, drills, and deployment to disaster or mass gathering events as part of their curriculum. These components serve to assess whether learning objectives have been met, i.e., whether performance has improved. (20–22).

Two-thirds of the programs are either nationally or internationally accredited. These are the programs that meet training standards about curriculum, methodology, credit system, etc. However, a more comprehensive international standardization of educational programs is essential for accreditation.

Most programs rely on tuition fees to cover instructional costs, in line with other training initiatives evaluated in previous studies (3, 23). Although scholarships and loans are available, financial support from governmental or non-governmental organizations would help expand these. Also, e-learning could be a way to reduce both direct and direct costs for trainees and institutions alike.

## Limitations

This study included training programs in disaster medicine. However, other programs, with a focus on emergency medicine, risk management, and biodefense, for example, may also take disaster medicine into consideration. We suggest that educational provision in these and other related fields be analyzed in further studies.

## Conclusion

This study identified 34 postgraduate programs in disaster medicine and highlighted a scarcity of training options in these fields. This applies especially to Asia, which is also the most vulnerable area. Half of the programs are delivered in the United States, which were listed in the top 10 countries for disaster occurrence in 2012. Educational provision must be strengthened in Asia and in low- and middle-income countries to enhance capacity building in the health management of disasters.

## Conflict of Interest Statement

The authors declare that the research was conducted in the absence of any commercial or financial relationships that could be construed as a potential conflict of interest.
